# Letter from the Editor in Chief

**DOI:** 10.19102/icrm.2022.130507

**Published:** 2022-05-15

**Authors:** Moussa Mansour


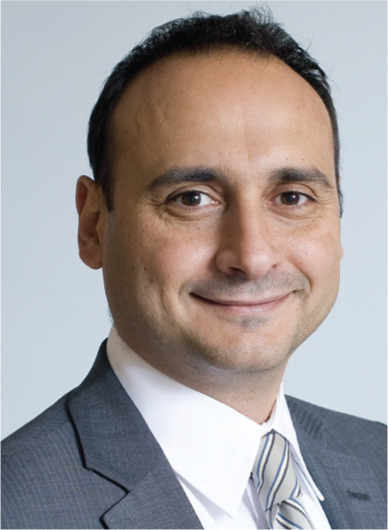


Dear readers,

At the recent annual scientific meeting of the Heart Rhythm Society in San Francisco, important science was presented, including 3 late-breaking randomized clinical trials likely to have significant impacts on clinical practice.

The first study was the ATLAS trial, which compared the safety of subcutaneous and transvenous implantable cardioverter-defibrillators (ICDs), presented by Jeff Healey, MD. Patients with inherited arrhythmia syndromes or at high risk for lead-related complications were randomized to a subcutaneous or transvenous ICD. The primary outcome was major lead-related complications occurring within 6 months of device implantation, including myocardial perforation, pneumothorax, lead dislodgement, subclavian venous thrombosis, and significant tricuspid insufficiency. Secondary endpoints included inappropriate shocks, failed appropriate clinical shocks, and sudden death. The results were impressive and demonstrated that, at 6 months of follow-up, the primary endpoint occurred in significantly fewer patients in the subcutaneous ICD group. No statistically significant difference was observed in the rate of inappropriate shocks or the rate of failed appropriate clinical shocks. This study is still ongoing, and additional results are expected regarding other outcomes, including the impact of ICD type on tricuspid valve regurgitation. The findings of this study corroborate those of previous studies regarding the safety of subcutaneous devices and will likely result in their expanded use.

The second study of note was the LBBP-RESYNC pilot trial presented by Fengwei Zou, MD, which compared the efficacy of left bundle branch (LBBB) pacing with cardiac resynchronization therapy (CRT) and biventricular pacing with CRT in 80 patients with non-ischemic cardiomyopathy and LBBB. The primary endpoint was the change in left ventricular ejection fraction at 3 and 6 months post-implantation, which was found to be significantly greater in patients who received LBBB pacing. The findings of this study also echoed those of similar studies presented at the conference, with all reporting a benefit of LBBB pacing compared to conventional biventricular pacing. This information will likely accelerate the recent trend that we have been witnessing in the field of pacing favoring the use of LBBB pacing.

The third study, which tackled a relatively new concept, was the Epicardial Ablation in Brugada Syndrome to Prevent Sudden Death trial. This prospective, single-center, randomized (2:1) study, presented by Giuseppe Ciconte, MD, enrolled patients with Brugada syndrome, prior ventricular fibrillation, and multiple appropriate ICD therapies. Patients were randomized to undergo epicardial ablation or not, and the primary endpoint was freedom from ventricular tachycardia/ventricular fibrillation recurrence during follow-up. The study demonstrated that ablation resulted in a significant reduction of ICD therapies compared to conventional medical therapy, suggesting that ablation is a viable option in this group of patients. This finding will likely lead to increased rates of ablation in this challenging patient population.

I look forward to reading the full publications of these studies, and I hope that you enjoy reading the articles in this issue of *The Journal of Innovations in Cardiac Rhythm Management*.

Sincerely,


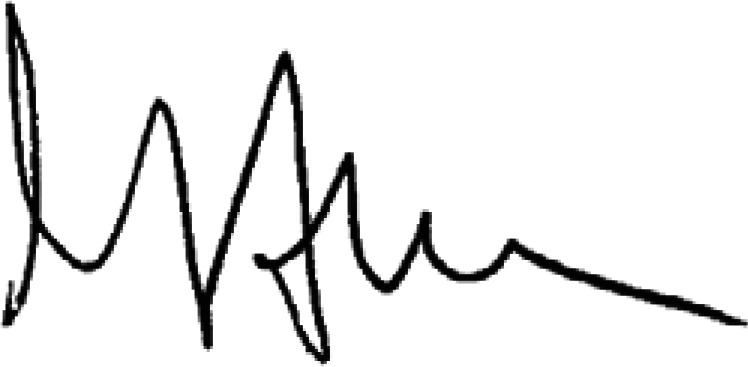


Moussa Mansour, md, fhrs, facc

Editor in Chief

*The Journal of Innovations in Cardiac Rhythm Management*

MMansour@InnovationsInCRM.com

Director, Atrial Fibrillation Program

Jeremy Ruskin and Dan Starks Endowed Chair in Cardiology

Massachusetts General Hospital

Boston, MA 02114

